# Hexagonal‐Close‐Packed Colloidal Crystals in *Glenea celestis* Beetles

**DOI:** 10.1002/smsc.202200114

**Published:** 2023-08-27

**Authors:** Alessandro Parisotto, Vinodkumar Saranathan, Ullrich Steiner, Bodo D. Wilts

**Affiliations:** ^1^ Adolphe Merkle Institute University of Fribourg Chemin des Verdiers 4 1700 Fribourg Switzerland; ^2^ Division of Sciences School of Interwoven Arts and Sciences Krea University 5655 Central Expressway Sri City Andhra Pradesh 517646 India; ^3^ Chemistry and Physics of Materials University of Salzburg Jakob-Haringer-Str. 2a 5020 Salzburg Austria

**Keywords:** biodiversity, biophotonics, colloidal crystals, hexagonal-close-packed, photonic crystals

## Abstract

Colloidal crystals reflecting interference‐based structural colors have been reported in many organisms, but many are yet to be accurately described. Herein, the bright, iridescent scales of green and blue *Glenea celestis* beetles are investigated using optical and ultrastructural techniques. The ultrastructural studies based on focused ion‐beam scanning electron microscopy and synchrotron small‐angle X‐ray scattering data reveal the origin of the colors to stem from previously undocumented hexagonal‐close‐packed colloidal crystals within their scales. The optical properties of various colloidal structures are investigated using full‐wave optical simulations. The results highlight the need for further research regarding the development of structural color in beetles.

## Graphical abstract

Through the use of various optical and structural characterization techniques, it is demonstrated that the colors of *Glenea celestis* beetles originate from hexagonal‐close‐packed (HCP) spheres found within their scales. Using full wave optical simulations, the optical properties of colloidal crystals with varying structures and fill fractions are compared. These findings have implications concerning the development and optical properties of natural photonic crystals.

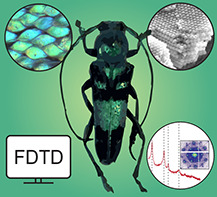

## 1 Introduction

Insects are among the most successful and diverse groups of organisms, partly due to their adaptability and ability to create complex architectures with limited materials. Various architectures are found in the colored scales of numerous insects,^[^ ^1^, ^2^, ^3^ ^]^ possessing nanostructured arrangements of chitin and air that create bright colors by the interference of incident light. These nanostructures serve as a bioinspired^[^ ^4^, ^5^, ^6^, ^7^, ^8^ ^]^ alternative to common human‐made inorganic pigments or dyes, which often contain compounds that are toxic to humans^[^ ^9^ ^]^ and marine life.^[^ ^10^ ^]^ The chitin in these organisms is a biodegradable, biocompatible, and non‐toxic material.^[^ ^11^ ^]^ Structural colors are seen as advantageous over pigmentary colors as they are highly tunable and nonbleaching.^[^ ^12^ ^]^ In insects, the structures capable of creating structural color are numerous and range in complexity from simple 1D thin films and multilayers^[^ ^13^, ^14^ ^]^ to more intricate 3D designs, including diamond^[^ ^15^, ^16^ ^]^ and gyroid networks.^[^ ^16^, ^17^, ^18^, ^19^ ^]^ As these structures possess periodic order on the length scale of visible light,^[^ ^20^ ^]^ they are called photonic crystals (PCs). In insects, these structural colors are usually distinguished from pigmented colors by their pronounced, visible iridescence, metallic appearance, and vibrant color. Amorphous or quasi‐ordered photonic crystals have recently gathered significant attention due to their ability to create angle‐independent structural colors by utilizing structures with short‐range but not long‐range order.^[^ ^21^, ^22^, ^23^, ^24^ ^]^ An example of such a structure exists in the scales of the beetle *Anoplophora graafi*,^[^ ^25^ ^]^ where chitin spheres of nearly uniform sizes assemble into a random close‐packed (RCP) arrangement. Although the arrangement of the spheres appears random, the nearest neighbor distance between the spheres is approximately constant, maintaining short‐range order in the structure. Color variations in the scales of this beetle arise from changes in sphere size and fill fraction (FF).^[^ ^25^ ^]^

Sphere‐based assemblies, called colloidal crystals, are also found in numerous crystalline geometries, such as face‐centered‐cubic (FCC), hexagonal‐close‐packed (HCP), or body‐centered‐cubic (BCC) arrangements. These structures have been reported in beetles such as *Pseudomyagrus waterhousei*,^[^ ^26^ ^]^ *Glenea celia*,^[^ ^27^ ^]^ *Anoplophora zonatrix*, as well as in numerous long‐horned beetles.^[^ ^16^ ^]^ While these studies have identified various morphologies, a complete 3D characterization is often missing, and local structure data are required to determine the colloidal structures accurately. In these studies, certain specimens possess both HCP and FCC stackings,^[^ ^27^ ^]^ while others are reported to possess mixtures of order and quasi‐ordered phases within each scale. These include the noniridescent scales of *P. waterhousei* and *A. zonatrix*, with quasi‐ordered morphologies alongside FCC and BCC structures, respectively.^[^ ^16^, ^26^ ^]^

In‐depth studies of sphere packing in insect scales are required because: i) single species/beetles can possess vastly different photonic structures,^[^ ^21^, ^22^, ^23^ ^]^ ii) colloidal crystals can fracture or disassemble when the outer cortex of these scales is removed for imaging, and iii) FCC and HCP colloidal packings are challenging to distinguish. While hard sphere assemblies weakly favor FCC stacking,^[^ ^28^, ^29^ ^]^ both phases often nucleate simultaneously and are usually found together.^[^ ^30^ ^]^ As a result, FCC and HCP structures are often interchangeably referred to as “opal” structures. There is, however, evidence that the two structures may possess different optical properties.^[^ ^31^ ^]^ HCP inverse opals and HCP‐stacked metal‐covered dielectric spheres possess larger photonic bandgaps than their FCC counterparts.^[^ ^32^, ^33^ ^]^ These enhanced bandgaps may offer an evolutionary advantage for beetles, and accurately identifying the structures within different insect PCs may provide insight into their structural and optical properties as well as their development.

Here, using optical and ultrastructural characterization techniques, including transmission small‐angle X‐ray scattering (SAXS) and scanning electron microscopy (SEM), we demonstrate that the colors of *Glenea celestis* beetles stem from ordered hexagonally‐close‐packed chitin spheres within their scales. Using finite‐difference time‐domain (FDTD) optical simulations, we explore the effect of crystal packing on their optical properties and discuss how these crystals are optimized to create brighter colors by comparing them to FCC‐packed structures.

## 2 Results

### 2.1 Optical Appearance

**Figure 1:**
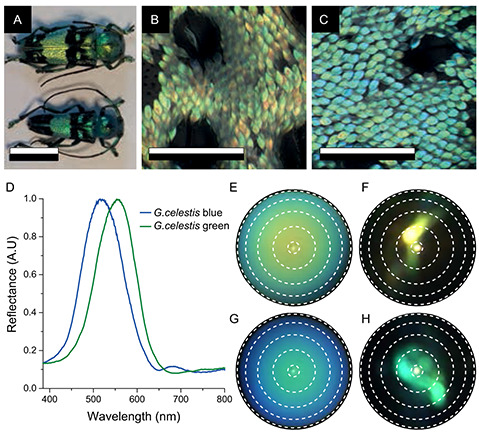
A) Habitus photograph of two *Glenea celestis* specimens. The elytra of these specimens consist of dark black spots and bright blue or green spots, where arrays of lenticular scales are found. B,C) Optical microscopy image of elytron scales of green (B) and blue (C) variants of *G. celestis*. The scales of both specimens are similar and resemble long lenticular teardrops, although the scales of the green specimen are slightly larger. D) Normalized reflectance spectra of various colored scales of green (*N* = 10) and blue specimens (*N* = 10), normalized to a white diffusive standard. E) Wide‐ and F) narrow‐angle *k*‐space images of a green‐orange *G. celestis* scale. G) Wide‐ and H) narrow‐angle *k*‐space image of a green‐blue *G. celestis* scale. The white rings represent scattering angles of 5°, 20°, 35°, 50°, and 65°. Scale bars: A) 1 cm; B,C) 200 μm.

*G. celestis* beetles are roughly 2–3 cm (sample size, *N* = 18) in length and have varying body colors ranging from turquoise‐blue to green. **Figure** **1**A shows two differently colored variants in green and blue—the extreme coloration variation observed in this species. While the photograph may indicate that color and size are related, our studies of 18 different specimens show no such relationship (Figure S1, Supporting Information). When observed by eye, both green and blue color variants appear brightly colored (Figure 1A). Light microscopy analysis reveals that the colors of these beetles originate from the colored, lenticular scales that adorn their otherwise black elytra (Figure 1B,C). The scales have an average width of 20 ± 2 μm in the blue variant (*N = *15) and 26 ± 1 μm in the green variant (*N = *15). The average scale length was 58 ± 5 μm in both variants (*N = *15).

Microphotospectrometry employing a custom‐adapted light microscope was used to investigate the spectral properties of the colored scales (see Experimental Section). Reflectance measurements of single scales show a prominent reflectance band with an average peak reflectance at 575 ± 17 nm for the green beetle scales (*N* = 10) and an average peak reflectance at 524 ± 17 nm for the turquoise‐blue beetle scales (*N* = 10), (Figure 1D). These pronounced reflectance peaks are indicative of a structural origin of the colors of the scales.^[^ ^34^ ^]^

The *k*‐space distribution of scattered light was imaged in both narrow‐ and wide‐angle illumination to visualize the scattering properties of the scales (Figure 1E–H). Narrow‐angle illumination (Figure 1F) of a green scale shows directional reflection from the scale. Wide‐angle illumination (Figure 1E) results in a varying color from orange, when viewed normal to the surface, to a green/blue hue at larger angles, which indicates iridescence.^[^ ^15^, ^34^ ^]^ *k*‐space images of a blue scale (Figure 1G,H) show similar, blue‐shifted behavior, as expected from the spectral measurements. These color variations were also observed on the scales on the wing. Variations in the local scale alignment and elytral curvature both contribute to variations in local elytral color (c.f. Figure 1B,C).

We further measured the transmission of single scales (*N* = 7) in a refractive index‐matching liquid with a refractive index of *n = *1.55, which is close to that of unpigmented, cuticular chitin.^[^ ^35^ ^]^ Figure S2, Supporting Information shows that light transmission reaches nearly 100%. This indicates the absence of light‐absorbing pigments and further confirms the structural origin of the scale colors.

### 2.2 Structural Analysis

**Figure 2:**
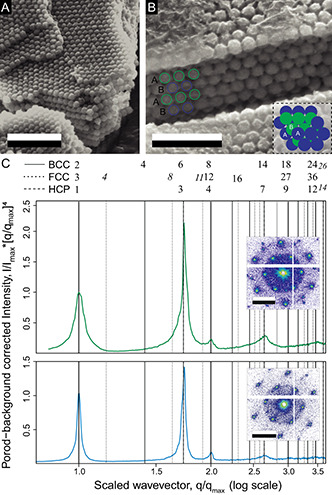
A) Electron microscopy images demonstrating the interior of a green *G. celestis* scale after its cortex has been removed with a plasma treatment. B) Electron microscopy image demonstrating the interior stacking structure of the colloidal structure found within the scales. Blue and green rings have been added to demonstrated the ABAB stacking feature of the structure. B,inset) Top view demonstrating an HCP packing structure for three planes. C) Porod‐background corrected azimuthally averages SAXS patterns of the scales of blue (top, inset) and green (bottom, inset) *G. celestis* beetles. The *q* factors and intensities are normalised to the first‐order peak. The vertical lines correspond to the expected Bragg peak positions of the HCP space group (dashed lines) (P6_3_/mmc), FCC (dotted lines), and BCC stackings (thin lines). Reflections common to two or more space groups are shown in gray or with thick black lines. The green and blue scales exhibit peak *q*‐factors of 0.0295 and 0.0327 nm^−1^, respectively. The average peak *q*‐factor was 0.0300 and 0.0321 nm^−1^ for the green and blue scales, respectively. The two insets in C) show the corresponding diffraction patterns. Scale bars: A) 25 μm; B) 2 μm; C) inset, 0.02 nm^−1^.

To explore the origin of the structural coloration within *G. celestis*, single scales were imaged by scanning electron microscopy. A physical plasma treatment first removed the continuous chitin cortex of the elytral scales,^[^ ^36^ ^]^ exposing an assembly of rough spheres (**Figure** **2**A). Although their sizes vary between different specimens, they are approximately 200 nm in diameter. The spheres are arranged in several layers, each possessing a periodic hexagonal in‐plane in both green and blue samples. The SEM data allow for measuring the characteristics of the PCs within the different scales, i.e., sphere size and nearest neighbor (NN) distance (Table S1 Supporting Information). The NN distance in the blue specimen was, on average, 20 nm smaller than that of the green specimen (Table S1, Figure S7A–C, Supporting Information). Figure S3B,C, Supporting Information demonstrates the distribution of the colloidal structure throughout the scales. Figure 2B offers a side view of the colloidal structure, which appears to possess an ABAB packing as is found for HCP colloids. We note that the scales do not appear to be close‐packed, and appear to be connected by small necking features, with average diameters of 45 ± 8 nm (*N = *20). These necking features are shown in Figure S6A,B, Supporting Information and they appear to affect the stability of structure, as they are found to be capable of holding the spheres in place even without underlying structures being in place (Figure S6C, Supporting Information). A focused ion beam (FIB) was used to cut across the sphere assemblies, exposing the scale cross sections. The cross section of a green scale (Figure 2) shows a non‐close‐packed assembly in which small necks with average diameters of 45 ± 8 nm (*N* = 20) connect the spheres. These necks seem to hold the spheres in place (Figure S6C, Supporting Information).

Since 2D SEM data are insufficient to characterize the 3D sphere packing, transmission SAXS measurements were performed on various scales from green and blue specimen. Figure 2C, inset shows a representative SAXS diffraction pattern of a green scale, with discrete Bragg spots exhibiting a hexagonal symmetry.^[^ ^16^, ^18^ ^]^ These spots are distributed in concentric hexagonal shells, characteristic of a single crystal structure.^[^ ^37^ ^]^ An azimuthal average of the SAXS patterns (Figure 2C), normalized with the peak structure factor (*q* = 0.0330 nm^−1^), allows indexing of the peaks of different scales (*N* = 20) (Figure 2C and S4, Supporting Information). These results reveal a hexagonally close‐packed (P6_3_/mmc) opal structure.^[^ ^16^ ^]^ Figure S4, Supporting Information shows additional peaks beyond the primary and secondary peaks in the Blue1 sample. These peaks are attributed to an SAXS pattern that averages over two adjacent domains possessing different in‐plane orientations. Comparing the peak positions with respect to the primary beam yields a value of 1.06, which is far from the 1.15 ratio expected for adjacent HCP and FCC domains.^[^ ^38^ ^]^ The lattice dimensions of the green and blue scales can be approximated by the Bragg equation $d=2\pi /q_{\text{pk}}$, where *d* represents the lattice spacing and *q*_pk_ is the primary peak. The lattice spacing was measured from the analysis of 10 SAXS azimuthally averaged spectra. Averaging the in‐plane lattice dimensions from all SAXS patterns yields a value of 210 ± 9 nm for the green scales and 196 ± 3 nm for the blue scales, closely matching the values obtained from the SEM images. The reflectance and SAXS peak measurements were correlated by the Maxwell–Garnet theory to obtain mean chitin FFs of 0.58 and 0.68 for blue and green scales, respectively.^[^ ^16^, ^37^ ^]^ Note that these FFs are too low for a close‐packed sphere arrangement, and we, therefore, term this structure hexagonal‐non‐close‐packed (H‐NCP).

### 2.3 FDTD Simulations

The effect of structure on the optical properties of various colloidal assemblies was studied with FDTD simulations. Idealized lattice structures were created, and their reflectance under normal incident light was simulated. Various parameters were varied to study the optics of different colloidal stackings. First, to compare the effect of packing structure on reflectivity, chitin spheres with a dimater of 220 nm were assembled into idealized HCP and FCC lattices. Their reflectance was measured under illumination along the [111] direction for the FCC PCs and along the [0001] direction for the HCP structures. This setup closely matches the findings of the SEM images in Figure 2A and S3A,B, Supporting Information. The spheres were also assembled into FCC and HCP assemblies with an NN distance of 220 nm with a smaller sphere size of 210 nm to design an NCP PC with an FF of 0.64, the average of the values determined from the experimental data (0.58–0.68).

**Figure 3:**
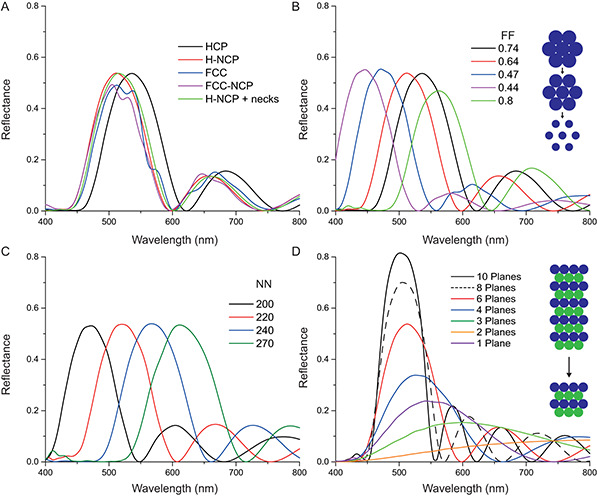
Finite‐difference time‐domain (FDTD) simulations of idealized colloidal crystal structures composed of equally sized spheres. A) Reflectance spectra from FDTD simulations of chitinous spheres assembled in idealised HCP and FCC arrangements. Spectra of non‐close‐packed (NCP) sphere assemblies are also shown. These were set up in either hexagonal (H‐NCP) or in FCC (FCC‐NCP) arrangements. The H‐NCP structures were also simulated with the bridging necks between the spheres (Figure 2B). B) Reflectance spectra of hexagonally arranged chitin spheres with FF‐values varying from 0.8 to 0.33. The FF was adjusted by changing the sphere diameter at a constant NN distance. The NN distance was kept constant in (A) and (B). C) Reflectance spectra of H‐NCP chitinous sphere assemblies with a fixed FF of 0.64 and varying NN distances. D) Simulated reflectance spectra of H‐NCP stacks with constant FF values and NN distances, where the number of crystal planes varied between 1 and 10. A schematic of the simulation setup is shown in Figure S5B, Supporting Information.

The HCP and FCC structures possess similar spectral responses, with a blueshift of 15 nm, a small decrease in peak reflectivity, and slight peak splitting of the FCC structure (**Figure** **3**A). The HCP structures show a large and clear reflectance band with a primary peak at approximately 535 nm and a substantially lower secondary peak near 690 nm. The FCC structure has a primary peak at 513 nm and a secondary peak at 670 nm. Note that the shape of the primary reflectance curves is similar to the experimentally measured green scale reflectances of Figure 1D. The experimental data show, however, no secondary reflectance peak for the green variants, and the secondary peak of the blue specimen is much less pronounced. Furthermore, the experimentally measured primary reflectance peak is at 575 ± 17 nm for the green beetle scales. The FCC and H‐NCP structures have similar spectral responses to their close‐packed counterparts, but their primary and secondary peaks are blueshifted by ≈5 nm. Small 45 nm‐wide bridging structures or “necks” were subsequently added between the spheres in the H‐NCP structures, similar to those seen in the SEM images of Figure 2B. Adding these bridging structures does not affect the reflectivity, apart from a very small 2 nm redshift. The electric field distribution within HCP, H‐NCP, and H‐NCP structures with necks was also compared (Figure S5A, Supporting Information). The electric fields concentrate near the surface of the spheres, and no enhanced scattering caused by the necks was observed.

The simulations were repeated for H‐NCP structures with a constant NN distance of 220 nm and FFs varying from 0.44 to 0.8 (Figure 3B), to determine the effect of the FF on the reflection spectra. An increase in FF leads to a redshift but does significantly change the reflectivity, except when the FF is raised above the close‐packed limit of 0.74, above which the reflectance decreases. The secondary peak brightness decreases with decreasing FF.

The effect of variations in NN distance on the reflectance of the structures was also tested in the NN range of 200–270 nm, with a constant FF of 0.64 (Figure 3C). This simulates the colloidal morphology in other insect species (e.g., in weevils^[^ ^21^, ^37^ ^]^), where color tuning is attributed to variations of both the NN distance and FF. As the observed secondary reflectance peak is unexpected, the number of simulated planes was varied to test whether Fabry–Pérot interferences are present, Figure 3D. The simulation results show a significant decrease in the reflectance with decreasing numbers of planes, accompanied by a red‐shift of the primary peak wavelength and an increase in the secondary peak width. These changes in the brightness and reflectance of the secondary peak strongly suggest that it indeed stems from Fabry–Pérot interference.

## 3 Discussion

Through a combination of ultrastructural and optical characterization techniques, we demonstrate that the bright colors of *G. celestis* beetles originate from hexagonal non‐close‐packed colloidal morphologies within their elytral scales (Figure 2, S4, Supporting Information; cf., ref. [16]). The SAXS scattering peaks of the scales correspond to those of a HCP (P6_3_/mmc) structure (Figure 2, S4, Supporting Information) with FFs ranging from 0.58 – 0.68. As these values are below the FF found for a perfectly close packed morphology, where FF = 0.74 the scale structure is referred to as non‐close‐packed (NCP). Based on our electron microscopy studies, we believe that the structure is able to maintain rigidity below a value of 0.74 with the use of necking structures, which are found between the adjacent spheres, Figure S6, Supporting Information. As is found in other species,^[^ ^21^, ^37^ ^]^ variations in the orientation, lattice parameter, and FF of the structures within the scales lead to variations in scale color from blue to orange (Figure S4 and S7, Supporting Information); however, as the scales are grouped together, these colors are found to average and the specimen appear either blue or green Figure 1A, S1, Supporting Information. A previous study on a similar species, *Glenea celia*, reports the presence of an NCP structure similar to that of *Glenea celestis*.^[^ ^27^ ^]^ However, as this work is mostly based on 2D projections, the reported structure is consistent with both FCC and HCP packings. The present study, therefore, presents the first conclusive report of a purely hexagonal colloid‐based structure within a living organism. Contrary to this study, our research finds that the scales of *G. celestis* consist of purely hexagonally packed colloidal nanostructures. Furthermore, the coherence lengths measured for the blue and green scales, 8.5 ± 2.2  and 10.2 ± 2.7 μm, are among the highest reported for ordered biophotonic nanostructures^[^ ^16^, ^18^, ^39^ ^]^ and are comparable to those achieved in synthetic opal assemblies.^[^ ^39^ ^]^

Previous works comparing the optical properties of HCP and FCC structures have shown differences in their band structure and resulting optical properties.^[^ ^31^, ^32^, ^33^ ^]^ However, these studies were limited to either four‐layer structures,^[^ ^31^ ^]^ dielectric spheres with metal shells,^[^ ^32^ ^]^ or inverse opals^[^ ^33^ ^]^ and are therefore not similar to the dielectric structures in the scales of *G. celestis*. We, therefore, performed FDTD simulations to understand how such structural differences would affect the optical properties of these natural colloidal assemblies. These simulations show that HCP‐ and FCC‐packed colloids possess similar reflectance spectra when oriented in the [0001] and [111] directions, respectively (Figure 3A). Although the reflectance spectra of the FCC structures do show slight spectral variations compared with HCP stacks, these are attributed to variations in the packing structure between FCC and HCP structures which may create a peak splitting. As the color variation between the scales on each beetle typically produces spectral averaging,^[^ ^40^ ^]^ these differences are likely not visible in vivo. However, the simulated HCP structure does more closely resemble the experimentally measured reflectance spectra Figure 1C. We note that though the simulated data possess secondary peaks, we attribute this phenomenon to Fabry–Pérot interference, as the shape and size of the secondary peaks are also altered by changing the number of planes of the simulated structure (Figure 3D). The spectral averaging found created by variations in scale orientation and color would likely eliminate this effect in vivo. The simulations further allow studying the effect of the FF on the optical properties of these non‐close‐packed (NCP) crystals. NCP structures have been reported to possess enhanced reflectivities compared to their close‐packed counterparts.^[^ ^41^, ^42^ ^]^ Contrary to these reports, our results indicate that decreasing the FF below 0.74 produces almost no change in the primary peak reflectance of the samples (Figure 3B). Changes in FF rather generate a color‐tuning effect, as has been reported in other beetle species with diamond‐structured photonic crystals.^[^ ^37^ ^]^ These results are explained by considering Bragg's law, as FCC structures oriented in the [111] direction possess identical lattice spacings, FFs, and refractive indices to HCP colloids oriented in the [0001] direction. Overall, we find the most significant contributor to reflectance and color saturation to be the number of planes within the structure (Figure 3D).

As the difference in the optical properties of HCP and FCC colloids is minimal, we suggest that the presence of a purely HCP structure within the scales of *G. celestis* is most likely related to the development of the structures themselves. The presence of a hexagonal structure strongly suggests that these structures do not assemble like hard spheres, as these are predominantly FCC or mixtures of FCC and HCP. Unfortunately, little is known about the in vivo formation of PCs in beetle scales, and no time‐dependent knowledge is available. We speculate that since butterflies have shared‐derived origins of insect scales from arthropod setae,^[^ ^43^ ^]^ the colloidal crystals of *G. celestis* may develop via the templated self‐assembly of an in‐folding smooth endoplasmic reticulum membrane, followed by deposition of chitin into the extracellular space.^[^ ^18^, ^19^, ^44^ ^]^ We note that in other self‐assembly processes, i.e., spherical micelle assembly in block‐copolymers, the HCP phase is found to be more stable.^[^ ^45^ ^]^ This may explain why the HCP phase is found within the scales of *G. celestis*, though further developmental studies are required to understand the assembly of PCs in beetles better. Performing such experiments may also offer valuable insight into the creation of the neck‐like structures, which offer significant structural stability, as is evidenced in Figure S3C, Supporting Information. As our FDTD simulations show that these necks do not deteriorate the reflectance of the photonic crystals (Figure S5A, Supporting Information, Figure 3A), we believe that these structures offer an interesting alternative to human‐made efforts to increase colloidal stability. Typically, stability is increased with the use of a high‐temperature sintering process^[^ ^46^ ^]^ that increases FF above 0.74, where it becomes detrimental to reflectance (Figure 3B). Furthermore, as these necking structures can be used to tune FF, they offer another mechanism with which to vary the colors of inverse opal structures created via templating methods.^[^ ^47^ ^]^

## 4 Conclusion

Through the combination of optical and ultrastructural studies with optical modeling, we demonstrate that the brightly colored scales of *G. celestis* beetles originate from purely HCP colloidal photonic crystals found within their scales. Our observations represent the first report of a purely HCP photonic crystal within a living organism. For these natural colloidal crystals, FDTD simulations reveal little difference between the optical properties of FCC and HCP colloidal photonic structures, and we find that decreases in FF do not affect the brightness of the structures, and therefore, we suggest that the presence of the HCP structure is the result of the formation process of these colloidal crystals—an area where more developmental research is necessary.

## 5 Experimental Section

5.1

5.1.1

##### Specimens

Specimens of *Glenea celestis* (Coleoptera: Cerambycidae: Lamiinae; Thomson, 1865; *N* = 17) were captured in Sulawesi, Indonesia, in July 2019 and purchased from a commercial retailer (www.thebugmaniac.com). Specimens were stored in dry and dark conditions.

##### Optical Characterization

Optical characterization was performed using a Xenon light source (Thorlabs SLS401; Thorlabs GmbH, Dachau, Germany) for both optical‐microscopy‐based measurements. Optical microscopy images were taken directly from the intact beetle using a Point Grey Grasshopper 3 USB3 camera (GS3‐U3‐28S5C‐C, Point Grey/FLIR Integrated Imaging Solutions Inc., Richmond, Canada) connected to a Zeiss AxioScope A1 microscope (Zeiss AG, Oberkochen, Germany). Spectroscopy measurements were performed with the Zeiss AxioScope A1 (Zeiss AG, Oberkochen, Germany), using a 20× objective lens (Zeiss Epiplan Neofluar, NA 0.6) and an optical fiber with a diameter of 230 μm that was mounted in a plane confocal to the image plane, resulting in a measurement spot size of ≈13 μm in diameter. *k*‐space images were captured after inserting a Bertrand lens (Zeiss 453671) into the optical path, using a (Zeiss Epiplan Neofluar, NA 0.9) 50× objective in the Zeiss AxioScope A1 microscope. In all spectral measurements, a white diffuser was used as a reference.

To test for scale pigmentation, single scales of *Glenea celestis* were scratched off their elytron onto microscope glass slides using a needle and transmission spectra of the single scales were measured in air and after application of a drop of refractive‐index‐matching oil with a refractive index of *n* = 1.55 (Cargille Labs, Series A) covered by a cover slip.

##### Ultrastructure Analysis

Ultrastructure analaysis was performed with a Tescan Mira3 LM field‐emission scanning electron microscopy (SEM) and a ThermoFisher Scios 2 DualBeam FIB‐SEM (FEI, Eindhoven, the Netherlands). Isolated scales were removed from the elytron with the use of a needle and subsequently placed onto aluminum stubs covered with conductive carbon tape. The outer cortex was then removed with the use of a 4:20 ratio of oxygen and argon plasma for 12–15 min using a PE‐100 RIE Benchtop Plasma Etching System (Plasma Etch Inc, Carson City, USA). Measurements of the nearest neighbor distances and sphere sizes were conducted using Fiji 2.0 software.^[^ ^48^ ^]^ Cross sections of single scales were prepared with the use of a FIB, where the center of the scales was milled with ThermoFisher Scios 2 FIB, using a voltage of 30 kV and milling current varying from 0.01 to 1 nA. The SEM images were taken at 52 with activated tilt correction.

##### Small‐Angle X‐ray Scattering

Small‐angle X‐ray scattering (SAXS) was performed on single scales that were scraped off of the elytron and placed onto an adhesive, 0.003 mm thick Kapton tape. Pinhole SAXS measurements were performed in transmission geometry at the ID‐02 beamline of the European Synchrotron Radiation Facility with an effective pinhole size of approx. 15 μm horizontal and 15 μm vertical. The data were processed with the use of SAXSutilities2 (https://www.saxsutilities.eu/).

##### Optical Simulations

Optical simulations were performed using Lumerical 2021 R2.5 (ANSYS Inc., Canonsburg, USA), a commercial FDTD solver. The studied volumes consisted of 1 μm cubes containing various assemblies of spheres that were assigned a constant refractive index of *n* = 1.55, which closely matches the refractive index of unpigmented, cuticular chitin^[^ ^35^ ^]^). The spheres were also given a slight imaginary index of *n* = 0.0001 to suppress Wood resonances. The overall simulation volume was 1.0 by 1.0 by 8.4 μm, with periodic boundary conditions on the sides and a perfectly matched layer beneath the simulated structure. The reflectance was measured from 400 to 800 nm. A schematic of the simulation setup is shown in Figure S5C, Supporting Information.

##### Statistical Analysis

Spectral measurements were taken from 10 different scales from blue and green specimens. The resulting data is plotted as the mean ± standard deviation (s.d.). The NN distances and sphere size measurements were measured from SEM images with around 15 measurements taken per sample with the use of Fiji (ImageJ 1.53c).^[^ ^48^ ^]^ The NN distance was also measured with the use of SAXS with 10 measurements taken from both green and blue scales, these are stated as mean ± s.d.

## Conflict of Interest

The authors declare no conflict of interest.

## Author Contributions

A.P. performed the measurements; B.D.W. conceptualized the work; B.D.W. and U.S. supervised the work and acquired funding; A.P. processed the experimental data, performed the analysis, drafted the manuscript, and designed the figures; V.S., U.S., and B.D.W. aided in interpreting the results and worked on the manuscript; all authors edited and approved the final version of the manuscript.

## Supporting information

Supplementary Material

## Data Availability

The data that support the findings of this study are available from the corresponding author upon reasonable request.

## References

[1] J. Sun , B. Bhushan , J. Tong , RSC Adv. 2013, 3, 14862.

[2] M. Srinivasarao , Chem. Rev. 1999, 99, 1935. 11849015 10.1021/cr970080y

[3] P. Vukusic , J. R. Sambles , Nature 2003, 424, 852. 12917700 10.1038/nature01941

[4] E. S. Goerlitzer , R. N. Klupp Taylor , N. Vogel , Adv. Mater. 2018, 30, 1706654. 10.1002/adma.201706654 29733481

[5] J. Xu , Z. Guo , J. Colloid Interface Sci. 2013, 406, 1. 23816221 10.1016/j.jcis.2013.05.028

[6] L. P. Biro , J.-P. Vigneron , Laser Photonics Rev. 2011, 5, 27.

[7] H. K. Raut , Q. Ruan , C. Finet , V. Saranathan , J. K. W. Yang , J. G. Fernandez , Adv. Mater. Interfaces 2022, 9, 2201419.

[8] Y. Liu , H. Wang , J. Ho , R. C. Ng , R. J. H. Ng , V. H. Hall-Chen , E. H. H. Koay , Z. Dong , H. Liu , C.-W. Qiu , J. R. Greer , J. K. W. Yang , Nat. Commun. 2019, 10, 4340. 31554803 10.1038/s41467-019-12360-w PMC6761189

[9] S. Jose , D. Joshy , S. B. Narendranath , P. Periyat , Sol. Energy Mater. Sol. Cells 2019, 194, 7.

[10] B. Lellis , C. Z. Fávaro-Polonio , J. A. Pamphile , J. C. Polonio , Biotechnol. Res. Innovation 2019, 3, 275.

[11] D. Elieh-Ali-Komi , M. R. Hamblin , Int. J. Adv. Res. 2016, 4, 411. PMC5094803 27819009

[12] S. Daqiqeh Rezaei , Z. Dong , J. You En Chan , J. Trisno , R. J. H. Ng , Q. Ruan , C.-W. Qiu , N. A. Mortensen , J. K. W. Yang , ACS Photonics 2021, 8, 18.

[13] D. G. Stavenga , Mater. Today: Proc. 2014, 1, 109.

[14] S. Yoshioka , T. Nakano , Y. Nozue , S. Kinoshita , J. R. Soc. Interface 2008, 5, 457. 17999945 10.1098/rsif.2007.1268 PMC2607392

[15] B. D. Wilts , K. Michielsen , H. De Raedt , D. G. Stavenga , J. R. Soc. Interface 2012, 9, 1609. 22188768 10.1098/rsif.2011.0730 PMC3367810

[16] V. Saranathan , A. E. Seago , A. Sandy , S. Narayanan , S. G. J. Mochrie , E. R. Dufresne , H. Cao , C. O. Osuji , R. O. Prum , Nano Lett. 2015, 15, 3735. 25938382 10.1021/acs.nanolett.5b00201

[17] K. Michielsen , D. G. Stavenga , J. R. Soc. Interface 2008, 5, 85. 17567555 10.1098/rsif.2007.1065 PMC2709202

[18] V. Saranathan , C. O. Osuji , S. G. Mochrie , H. Noh , S. Narayanan , A. Sandy , E. R. Dufresne , R. O. Prum , Proc. Natl. Acad. Sci. USA 2010, 107, 11676. 20547870 10.1073/pnas.0909616107 PMC2900708

[19] B. D. Wilts , B. A. Zubiri , M. A. Klatt , B. Butz , M. G. Fischer , S. T. Kelly , E. Spiecker , U. Steiner , G. E. Schröder-Turk , Sci. Adv. 2017, 3, e1603119. 28508050 10.1126/sciadv.1603119 PMC5406134

[20] D. Joannopoulos , Photonic Crystals: Molding the Flow of Light, 2nd ed., Princeton University Press, Princeton, NJ, USA 2008.

[21] A. Parisotto , U. Steiner , A. A. Cabras , M. H. Van Dam , B. D. Wilts , Small 2022, 18, 2200592. 10.1002/smll.202200592 35426236

[22] K. Djeghdi , U. Steiner , B. D. Wilts , Adv. Sci. 2022, 9, 2202145. 10.1002/advs.202202145 PMC9475527 35852001

[23] C. Pouya , D. G. Stavenga , P. Vukusic , Opt. Express 2011, 19, 11355. 21716365 10.1364/OE.19.011355

[24] E. Bermúdez-Ureña , C. Kilchoer , N. P. Lord , U. Steiner , B. D. Wilts , iScience 2020, 23, 101339. 32688285 10.1016/j.isci.2020.101339 PMC7371903

[25] B. Q. Dong , X. H. Liu , T. R. Zhan , L. P. Jiang , H. W. Yin , F. Liu , J. Zi , Opt. Express 2010, 18, 14430. 20639928 10.1364/OE.18.014430

[26] P. Simonis , J. P. Vigneron , Phys. Rev. E 2011, 83, 11908. 10.1103/PhysRevE.83.011908 21405714

[27] J. W. Galusha , L. R. Richey , M. R. Jorgensen , J. S. Gardner , M. H. Bartl , J. Mater. Chem. 2010, 20, 1277.

[28] P. G. Bolhuis , D. Frenkel , S.-C. Mau , D. A. Huse , Nature 1997, 388, 235.9230429

[29] L. Woodcock , Nature 1997, 385 141.

[30] I. Sanchez-Burgos , E. Sanz , C. Vega , J. R. Espinosa , Phys. Chem. Chem. Phys. 2021, 23, 19611. 34524277 10.1039/d1cp01784e

[31] X. Checoury , S. Enoch , C. López , A. Blanco , Appl. Phys. Lett. 2007, 90, 161131.

[32] W. Zhang , C. T. Chan , P. Sheng , Opt. Express 2001, 8, 203. 19417805 10.1364/oe.8.000203

[33] K. Busch , S. John , Phys. Rev. E 1998, 58, 3896.

[34] B. D. Wilts , H. L. Leertouwer , D. G. Stavenga , J. R. Soc. Interface 2009, 6, S185. 18782721 10.1098/rsif.2008.0299.focus PMC2586095

[35] H. L. Leertouwer , B. D. Wilts , D. G. Stavenga , Opt. Express 2011, 19, 24061. 22109431 10.1364/OE.19.024061

[36] Y. Chang , Y. Ogawa , G. Jacucci , O. D. Onelli , H. Y. Tseng , S. Vignolini , Adv. Opt. Mater. 2020, 8, 2000432.

[37] B. D. Wilts , V. Saranathan , Small 2018, 14, 1802328. 10.1002/smll.201802328 30112799

[38] J. X. Yang , H. L. Zhao , H. R. Gong , M. Song , Q. Q. Ren , Sci. Rep. 2018, 8, 1992. 29386540 10.1038/s41598-018-20257-9 PMC5792657

[39] V. Saranathan , S. Narayanan , A. Sandy , E. R. Dufresne , R. O. Prum , Proc. Natl. Acad. Sci. 2021, 118, e2101357118. 34074782 10.1073/pnas.2101357118 PMC8201850

[40] A. E. Seago , P. Brady , J.-P. Vigneron , T. D. Schultz , J. R. Soc. Interface 2009, 6, S165. 18957361 10.1098/rsif.2008.0354.focus PMC2586663

[41] L. Wang , Q. Yan , X. S. Zhao , J. Mater. Chem. 2006, 16, 4598.

[42] Z. Zhou , Q. Yan , Q. Li , X. S. Zhao , Langmuir 2007, 23, 1473. 17241075 10.1021/la062601v

[43] A. E. Seago , R. Oberprieler , V. K. Saranathan , Integr. Comp. Boil. 2019, 59, 1664. 10.1093/icb/icz040 31093648

[44] H. Ghiradella , J. Morphol. 1989, 202, 69. 29865680 10.1002/jmor.1052020106

[45] L.-T. Chen , C.-Y. Chen , H.-L. Chen , Polymer 2019, 169, 131.

[46] H. Miguez , F. Meseguer , C. Lopez , A. Blanco , J. S. Moya , J. Requena , A. Mifsud , V. Fornes , Adv. Mater. 1998, 10, 480. 21647983 10.1002/(SICI)1521-4095(199804)10:6<480::AID-ADMA480>3.0.CO;2-Y

[47] H. K. Raut , H. Wang , Q. Ruan , H. Wang , J. G. Fernandez , J. K. W. Yang , Nano Lett. 2021, 21, 8602. 34662137 10.1021/acs.nanolett.1c02483

[48] J. Schindelin , I. Arganda-Carreras , E. Frise , V. Kaynig , M. Longair , T. Pietzsch , S. Preibisch , C. Rueden , S. Saalfeld , B. Schmid , J.-Y. Tinevez , D. J. White , V. Hartenstein , K. Eliceiri , P. Tomancak , A. Cardona , Nat. Methods 2012, 9, 676. 22743772 10.1038/nmeth.2019 PMC3855844

