# Supplementary material for: Hexagonal‐Close‐Packed Colloidal Crystals in Glenea celestis Beetles
Source: Small Sci. 2023 Aug 27;3(10):2200114. doi: 10.1002/smsc.202200114 (PMC11935839; doi:10.1002/smsc.202200114)
Supplement: Supplementary file 1 — Supplementary Material [file SMSC-3-2200114-s001.pdf]

Supporting Information

for

Hexagonal-close-packed colloidal crystals in *Glenea celestis* beetles

Alessandro Parisotto[1], Vinodkumar Saranathan[2], Ullrich Steiner[1], and Bodo D. Wilts[1,3,*]

[1]Adolphe Merkle Institute, Chemin des Verdiers 4, University of Fribourg, 1700 Fribourg, Switzerland
[2]Division of Sciences, School of Interwoven Arts and Sciences, Krea University, 5655 Central Expressway, Sri City, Andhra Pradesh 517646, India
[3]Chemistry and Physics of Materials, University of Salzburg, Jakob-Haringer-Str. 2a, 5020 Salzburg, Austria
[*]Corresponding author, e-Mail: bodo.wilts@plus.ac.at

Supplementary Figures

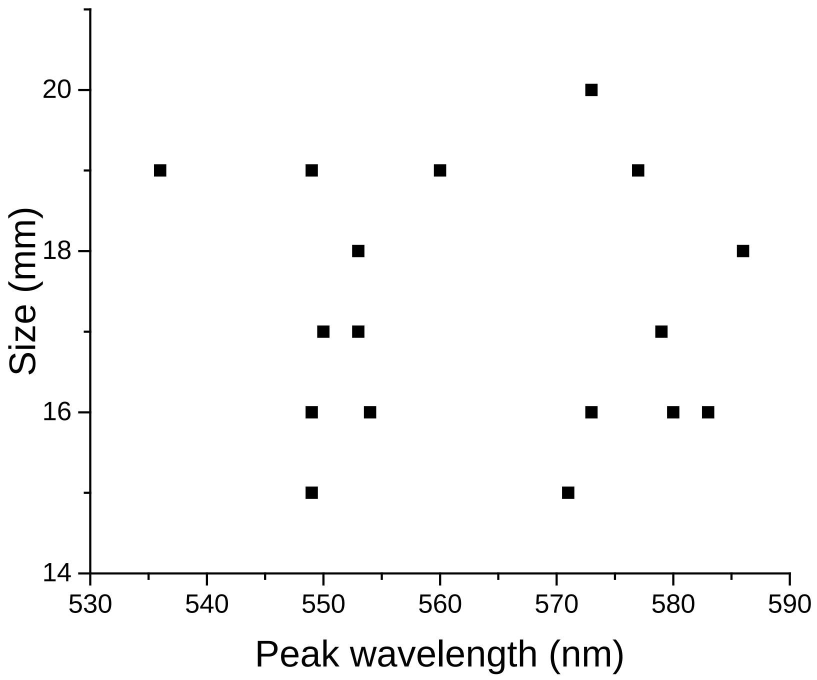

Figure S1: Beetle size (head to pygidium) and average reflected wavelength measured for 17 different *G. celestis* specimen.

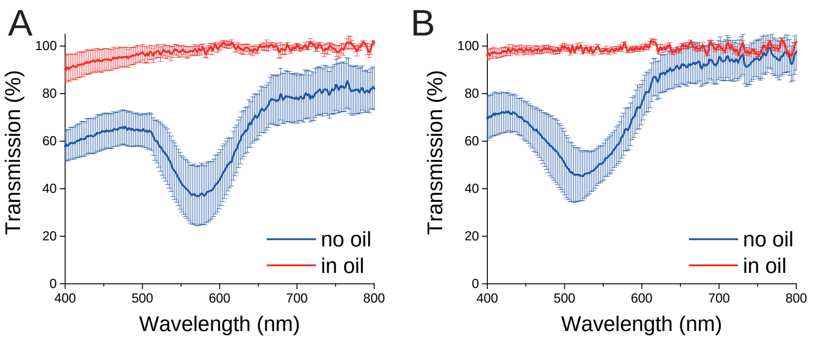

Figure S2: A) Transmission spectra of *G. celestis* blue scales (left) both with and without filling with a refractive index matching oil (n=1.55). B) Transmission spectra of *G. celestis* green scales (left) both with and without filling with a refractive index matching oil (n=1.55). Each measurement is averaged from 5 – 7 spectra, the error is the s.d.

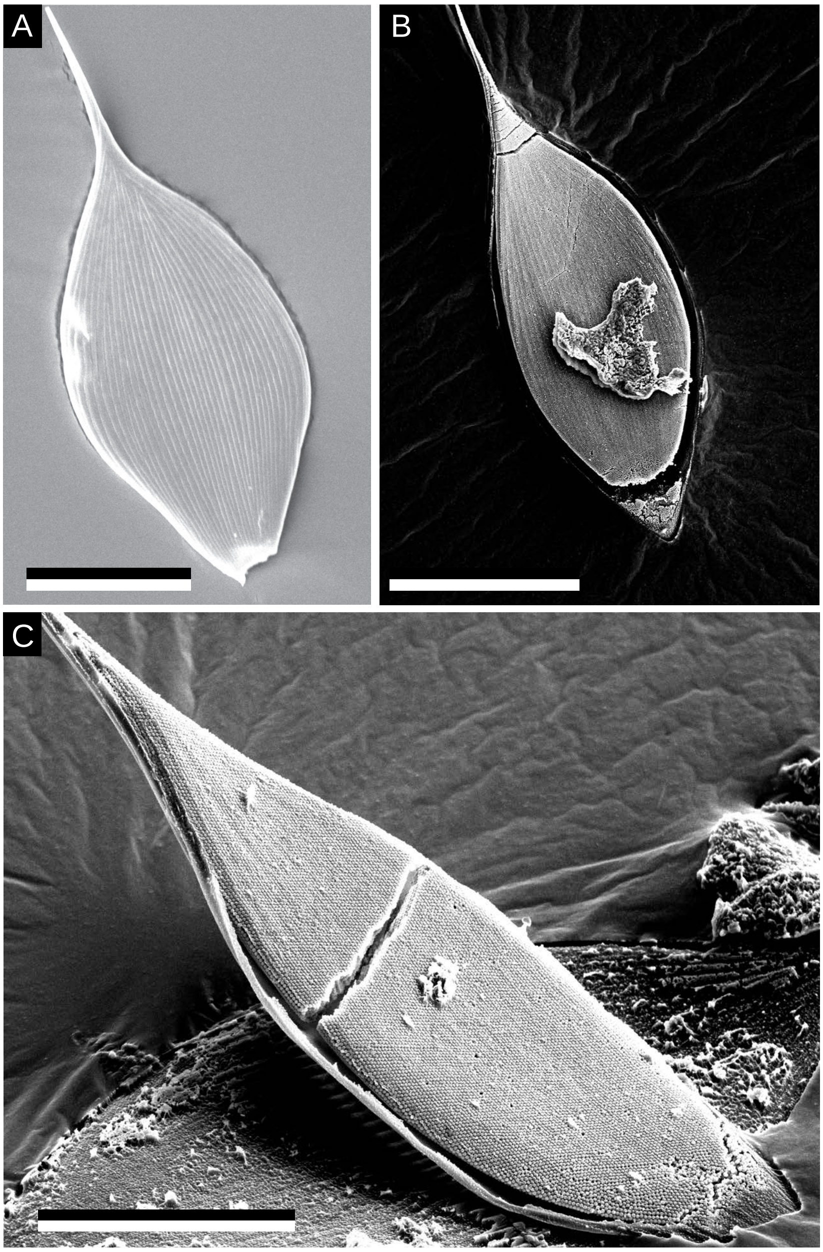

Figure S3: A) Top view SEM image of a *G. celestis* scale before plasma treatment. B,C) Electron Microscope images of two scales which have had their cortex removed via plasma treatment. Both images offer a greater view of the distribution of the colloidal structure throughout the scales. DScale bars: A,B,C) 25 µm

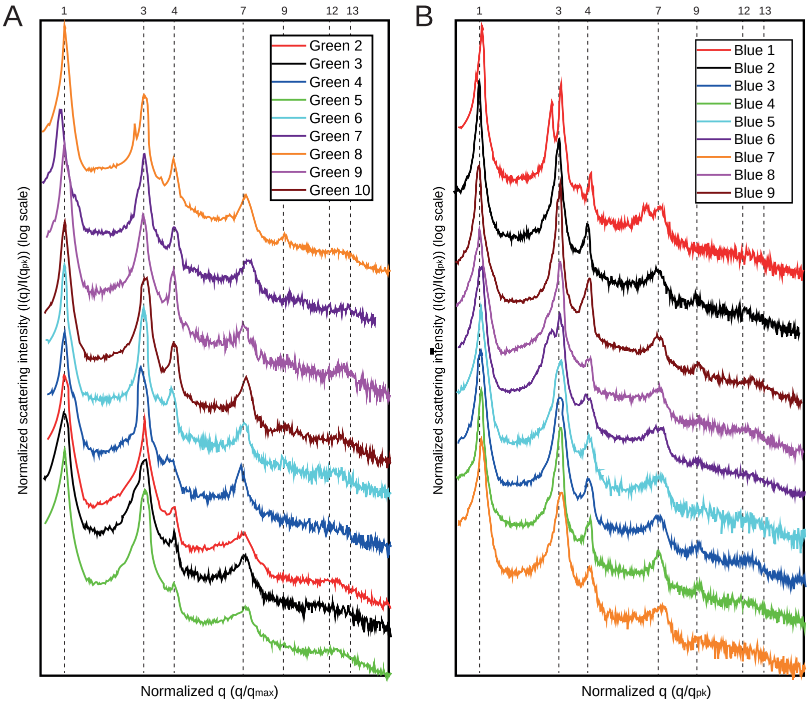

Figure S4: A) Azimuthal average of the SAXS patterns of various scales from green *G. celestis* beetles on a log-log scale. Both the q factor and intensity have been normalized to the maximal peak of each spectrum. The vertical lines correspond to the expected Bragg peak positional ratios for a hexagonal close-packed 3D opal structure. B) Azimuthal average of the SAXS patterns of various scales from blue *G. celestis* beetles on a log-log scale.

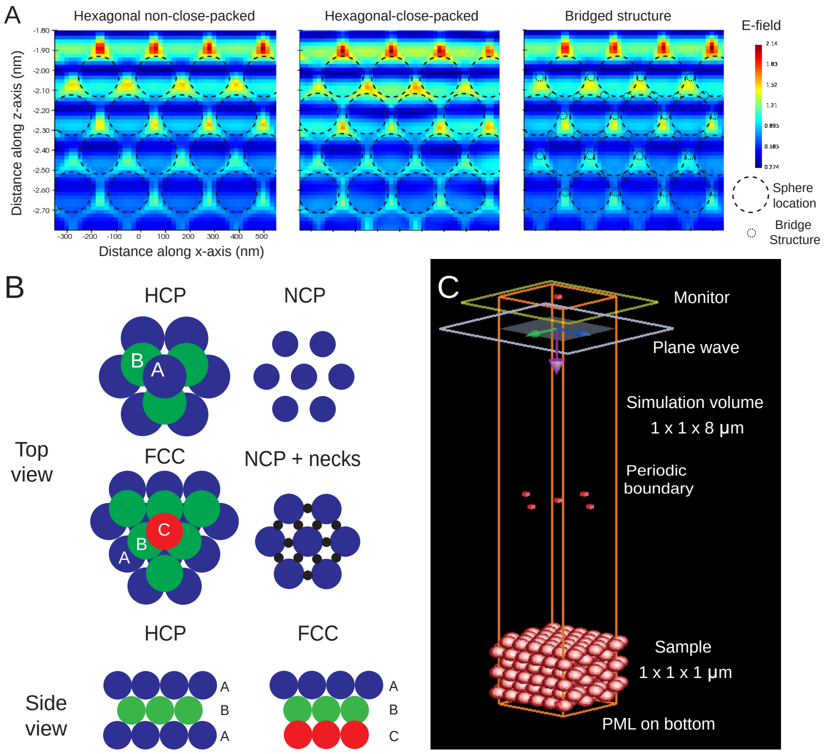

Figure S5: A) Electric field distribution from the top to the bottom of an HCP, Hex NCP and Hex NCP structure with 45 nm sphere bridges at a wavelength of 500 nm. The field distribution is measured along the axis with which light impedes the photonic crystal. B) Simple schematics demonstrating the different structures and labels used within the paper, side view and top view are included to more accurately distinguish HCP and FCC stacking structures. C) Schematic of the setup used for FDTD simulations.

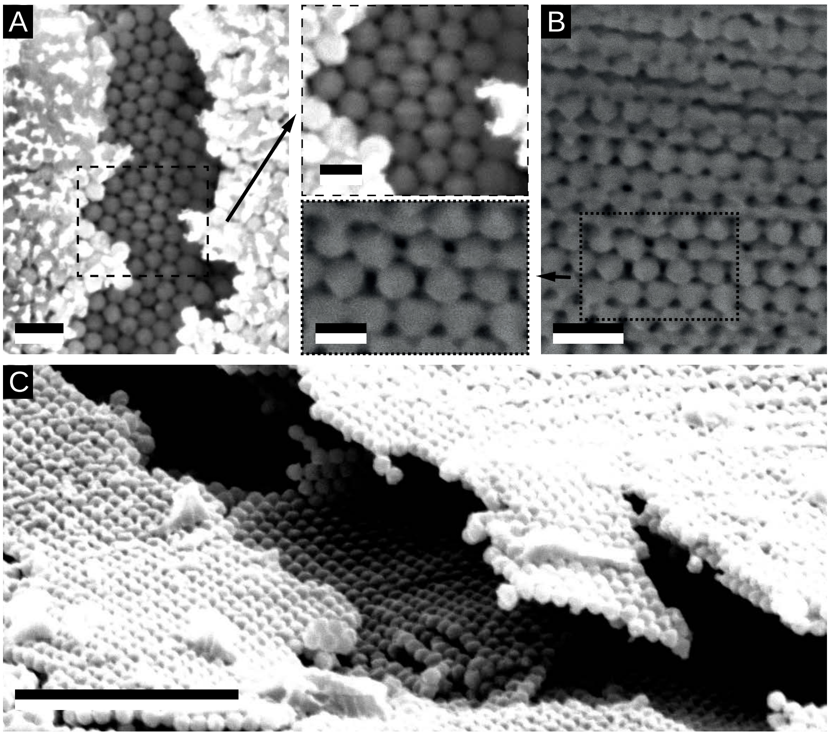

Figure S6: A) Top view SEM image of a *G. celestis* scale after plasma treatment, the cortex has opened, revealing the inner structure within the scales. B) Electron Microscope images of a FIB cross-section of a scale. C) Electron microscope image of a *G. celestis* scale post plasma treatment. The scale has cracked open and the colloidal structure has been split. Here, it can be seen that the spheres appear connected via some structures. A,B) 500 nm; A,B insets) 250 nm; C) 2.5 µm.

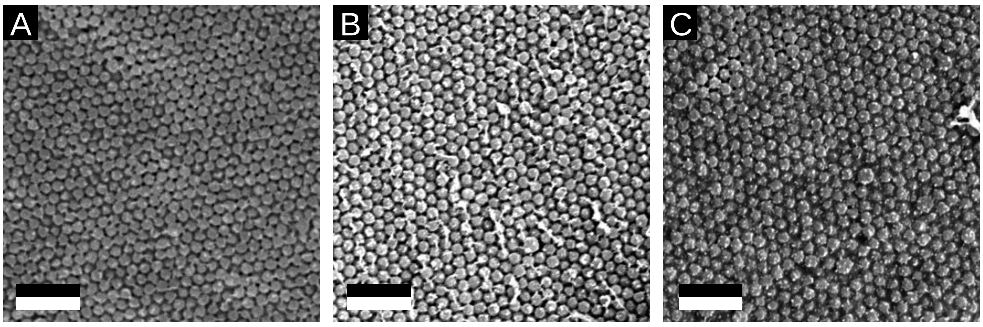

Figure S7: Top view images of *G. celestis* after plasma treatment from blue (A), green (B) and orange (C) scales, the average NN distance for these scales are 190 ± 10 220 ± 10 and 230 ± 10 nm respectively. Scale bars: A,B,C) 1 µm.

Supplementary Tables

Table 1: Spectroscopic and structural parameters of *G. celestis* specimen.

**Specimen**	***G. celestis* green**	***G. celestis* blue**
Sphere diameter (nm)	200 ± 10	170 ± 10
Nearest neighbour distance (nm)	220 ± 10	190 ± 10
Average peak wavelength (nm)	580 ± 20	520 ± 20
Average scale area (μm^2)	1300 ± 200	1000 ± 100
Average scale length (μm)	60 ± 5	60 ± 2
Average scale width (μm)	26 ± 1	20 ± 2
